# COVID-19 surveillance: Large decrease in clinical notifications and epidemiological investigation questionnaires for laboratory-confirmed cases after the 2nd epidemic wave, Portugal March 2020–July 2021

**DOI:** 10.3389/fpubh.2023.963464

**Published:** 2023-03-09

**Authors:** Vasco Ricoca Peixoto, André Vieira, Pedro Aguiar, Alexis Sentis, Carlos Carvalho, Daniel Rhys Thomas, Alexandre Abrantes, Carla Nunes

**Affiliations:** ^1^NOVA National School of Public Health, Public Health Research Centre, Comprehensive Health Research Center, CHRC, NOVA University Lisbon, Lisbon, Portugal; ^2^Epidemiology Department, Epiconcept, Paris, France; ^3^Unit for Multidisciplinary Research in Biomedicine, Abel Salazar Institute of Biomedical Sciences, University of Porto, Porto, Portugal; ^4^Communicable Disease Surveillance Centre, Public Health Wales, Cardiff, United Kingdom

**Keywords:** COVID-19, completeness, surveillance attribute, epidemic surveillance, surveillance system evaluation, notifications

## Abstract

**Introduction:**

In Portugal, COVID-19 laboratory notifications, clinical notifications (CNs), and epidemiological investigation questionnaires (EI) were electronically submitted by laboratories, clinicians, and public health professionals, respectively, to the Portuguese National Epidemiological Surveillance System (SINAVE), as mandated by law. We described CN and EI completeness in SINAVE to inform pandemic surveillance efforts.

**Methods:**

We calculated the proportion of COVID-19 laboratory-notified cases without CN nor EI, and without EI by region and age group, in each month, from March 2020 to July 2021. We tested the correlation between those proportions and monthly case counts in two epidemic periods and used Poisson regression to identify factors associated with the outcomes.

**Results:**

The analysis included 909,720 laboratory-notified cases. After October 2020, an increase in the number of COVID-19 cases was associated with a decrease in the submissions of CN and EI. By July 2021, 68.57% of cases had no associated CN nor EI, and 96.26% had no EI. Until January 2021, there was a positive correlation between monthly case counts and the monthly proportion of cases without CN nor EI and without EI, but not afterward. Cases aged 75 years or older had a lower proportion without CN nor EI (aRR: 0.842 CI95% 0.839–0.845). When compared to the Norte region, cases from Alentejo, Algarve, and Madeira had a lower probability of having no EI (aRR;0.659 CI 95%0.654–0.664; aRR 0.705 CI 95% 0.7–0.711; and aRR 0.363 CI 95% 0.354–0.373, respectively).

**Discussion:**

After January 2021, CN and EI were submitted in a small proportion of laboratory-confirmed cases, varying by age and region. Facing the large number of COVID-19 cases, public health services may have adopted other registry strategies including new surveillance and management tools to respond to operational needs. This may have contributed to the abandonment of official CN and EI submission. Useful knowledge on the context of infection, symptom profile, and other knowledge gaps was no longer adequately supported by SINAVE. Regular evaluation of pandemic surveillance systems' completeness is necessary to inform surveillance improvements and procedures considering dynamic objectives, usefulness, acceptability, and simplicity.

## Introduction

Facing the large number of COVID-19 cases public health services may have adopted other registry strategies including new surveillance and management tools to respond to operational needs.

In Portugal, the established National Epidemiological Surveillance System (SINAVE) (1, 2) was updated to include COVID-19 laboratory notifications (LNs), submitted by the laboratories, clinical notifications (CNs), submitted by the clinicians or public health professionals in local-level public health units, and epidemic investigation (EI) questionnaires, submitted by the public health professionals or under their supervision, according to EU regulations and national law. These components are mandatory by law (2), which states that CN must be submitted within 24 h of diagnosis (2) and the EI and contact tracing must be initiated within 24 h of acknowledgment of the case by the public health services. LN, CN, and EI must be recorded in SINAVE digital platform (3). COVID-19 management teams at the local public health services had to verify LN, ensure the existence of a CN, submit an EI questionnaire in SINAVE, and initiate contact tracing. If a CN was not submitted by a clinician, public health services should guarantee their submission with the necessary information (3).

Clinical notifications include symptoms, comorbidities, outcomes, and basic epidemiological information. EI could only be registered if a CN was submitted. EI registries included both CN information and epidemiological information regarding the context of infection, epidemiological link, association with outbreaks, travel history, workplace, and activities; however, they did not allow for the recording of contact tracing lists and there was no national system for recording of contact tracing activities and results until the development of TRACE COVID-19 (4). This platform was created to support contact tracing and follow-up of contacts (including self-reporting of symptoms), case management at home, symptom recording, and follow-up by clinicians. This platform was regularly updated to include more functions throughout the pandemic. CN and EI records could be updated or corrected by Health Authorities at the local level (in public health units), regional level (Health Regional Administrations' Public Health Department), and national level (by the Portuguese Directorate-General of Health—DGS).

The importance of surveillance system's attributes evaluation, such as acceptability, simplicity, internal completeness, sensitivity, internal data validity, timeliness, usefulness, and others, resurfaced with COVID-19. The performance of surveillance systems considering these attributes is relevant because they determine the following: the quality of information, the behavior of data providers, and data quality. This will have an impact on research and routine surveillance to inform strategic decision-making.

We identified few reported COVID-19 surveillance system evaluations for COVID-19 (5–7) focusing on the attributes proposed by the European Centre for Disease Prevention and Control (ECDC) (8) and Centers for Disease Control and Prevention (CDC) (9). Some focus on laboratory surveillance but evaluate the internal completeness of demographic and epidemiological variables (10). We found no reports of internal completeness of submission of different components of surveillance (clinical notification, epidemiological investigation questionnaires/contact tracing) and their changes over time by age and region. The ECDC has issued guidance for surveillance system evaluation and data quality monitoring that stresses the importance of evaluating system internal completeness through time in face of changing pandemic context, guidelines, and evolving surveillance systems and tools (8).

In Portugal, after October 2020, there was a large increase in cases that made it necessary for public health services to adapt and quickly escalate capacity for collecting information and submitting clinical notifications (CNs), epidemiological investigation (EI) questionnaires in SINAVE, and for contact tracing.

During this high-incidence period, many healthcare workers and professionals from other sectors, including military personnel, were mobilized to aid in epidemiological investigation activities and contact tracing.

The changes in procedures and demand in different pandemic phases, the different strategies to cope with increased demand in different regions, and the emergence of parallel contact tracing tools that worked as concurrent surveillance systems with operational tools for COVID-19, triggered the importance of evaluating the completeness of SINAVE in an evolving epidemic context.

In this study, we aimed at describing the completeness of SINAVE regarding the submission of clinical notification and epidemiological investigation questionnaires and to understand the associated factors to inform future evaluation needs and potential risks in pandemic surveillance.

## Methods

### Study design and data sources

We used a database including all COVID-19 laboratory-confirmed cases in Portugal from 3 March 2020 until 16 July 2021. This database is compiled by the Portuguese Directorate General of Health (DGS) and includes all first positive laboratory notifications for each patient. It also contains information related to CN and EI questionnaires for each LN if they were submitted. EI questionnaires can only be recorded and submitted if a clinical notification is previously submitted.

We described the proportion of cases without CN nor EI and without EI overall (regardless of CN) in time, place, and person. We identified the factors associated with these outcomes in multivariable analysis using Poisson regression and presented adjusted relative risks, 95% confidence intervals, and *p*-value.

Finally, we calculated the Pearson correlation coefficient and respective *p*-value between the number of cases in each month and the proportion of cases with EI questionnaires by region in two different periods (before and after January 2021, the largest peak of hospitalizations by COVID-19) and presented coefficient variation from one period to another.

### Outcomes

The analyzed outcomes were as follows: laboratory-confirmed cases without CN nor EI; laboratory-confirmed cases without EI overall (regardless of submission of clinical notification).

### Associated factors

We described both the outcomes by sex, age, and health region in each month. Age was categorized into four groups (<25; 25–49; 50–74; ≥75). Region was categorized according to the county of occurrence and, when not available, the county of residence was used.

### Statistical analysis

We calculated the proportion of cases for each outcome in the different health regions and plotted the monthly proportion of LN without CN nor EI and without EI overall including the number of cases in each month by region for visual comparison of trends in completeness and case numbers. We calculated the proportion of each outcome by month for different age groups.

We conducted univariable and multivariable analyses for both outcomes for each category of the associated factors and calculated prevalence by category and adjusted relative risks (aRRs), 95% confidence intervals (95%CIs), and respective *p*-values.

Statistical analysis was conducted in Stata (version 14, StataCorp, College Station, Texas, US). All analyses used 95% CI and considered a *p* < 0.05 as statistically significant.

## Results

All positive 909,720 laboratory notifications (LNs) between 3 March 2020 and 16 July 2021 were included in the analysis. Of those, 486,451 (53.47%) had no CN nor EI and 762,512 (83.82%) had no EI overall.

**Figure 1:**
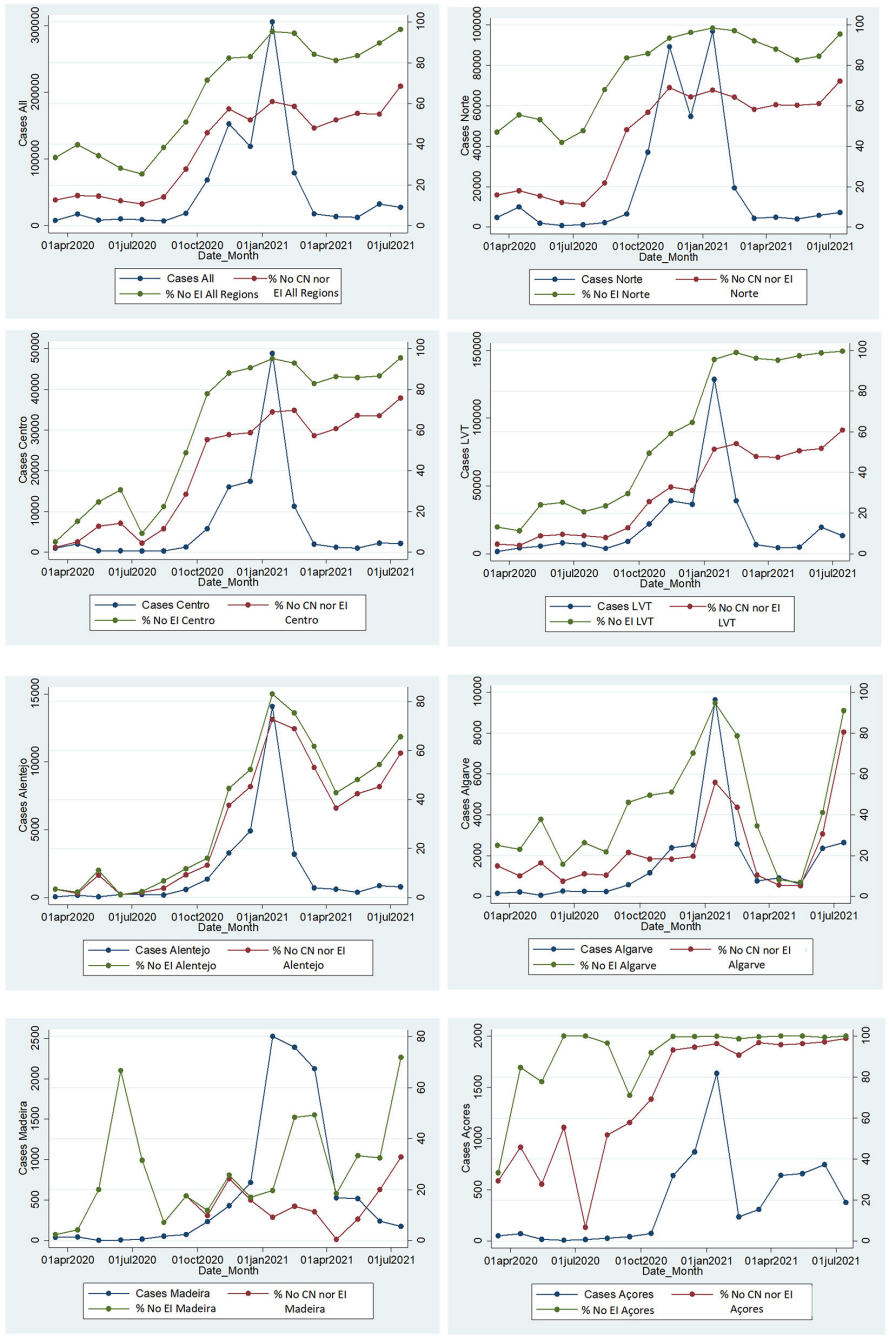
Monthly laboratory-confirmed cases and proportion of cases without clinical notification or epidemic investigation questionnaire submitted (No CN nor EI), and proportion of cases without EI questionnaires overall (No EI) by month in each Region and in Portugal, SINAVE, Portugal March 2020–July 2021. Cases: A total number of cases with a laboratory notification. No CN nor EI: cases without clinical notification and without EI questionnaire. No EI: cases without a submitted epidemic investigation questionnaire overall (regardless of clinical notification).

We observed that, with a rise in cases starting in October 2020, in most regions, there was a large increase in the proportion of cases with no CN nor EI and without EI overall (Figure 1).

Even before the large increase in case numbers after October 2021, some regions had higher proportions of cases without EI such as North, LVT, and Center.

After stringent control measures in January to contain the spread, there has been a steep reduction in cases in all regions, with a slower reduction in Madeira. Even though in regions such as Algarve and Alentejo, there was a reduction in the proportion of cases without CN nor EI and without EI overall, in most regions, these proportions remained above 60 and 80%, respectively.

Age ≥75 had lower proportions of cases without clinical notification nor EI questionnaires and without EI overall. However, differences between age groups were larger for the proportions of cases without CN nor EI. The EI questionnaire was missing in high proportions of cases in all age groups, and even though there was a mild reduction in February, March, and April 2021, there was a rise in all age groups reaching levels close to 90% for those above 75 and close to 100% for other age groups in the last analyzed month.

**Figure 2:**
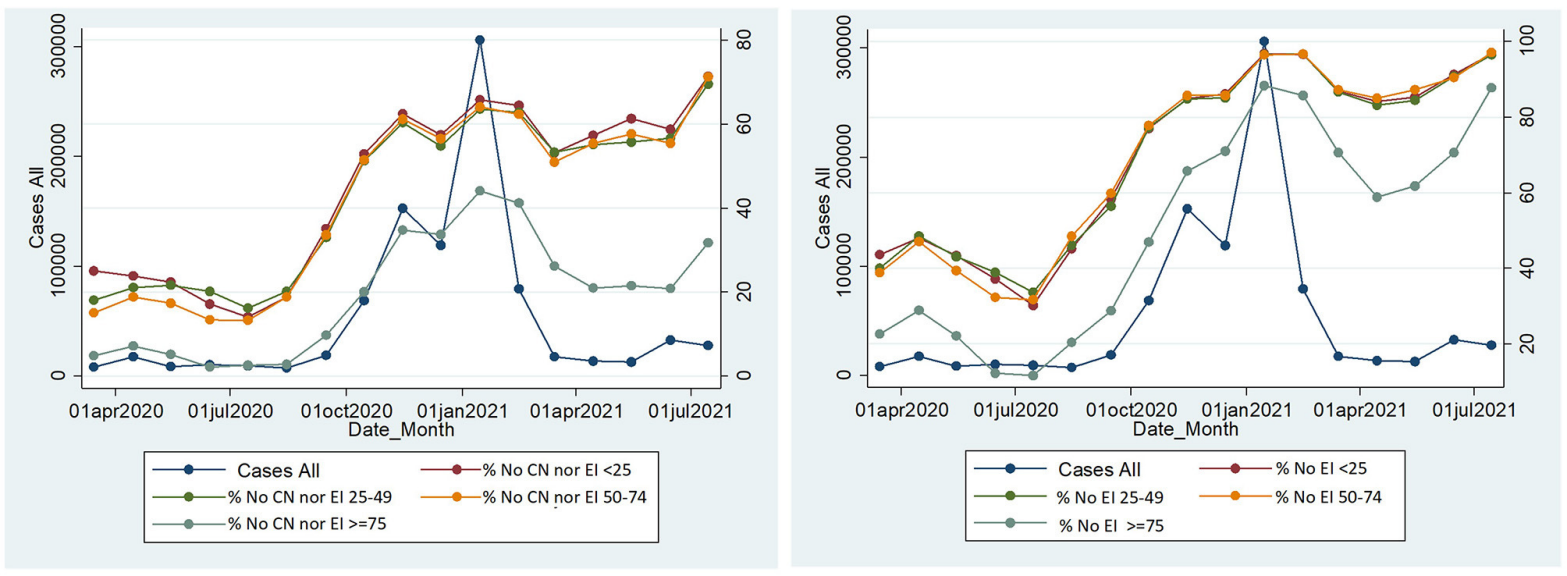
Monthly number of cases and proportion of cases without CN nor EI by age group (left) and Monthly number of cases and proportion of cases without EI overall (regardless of CN) in each month, by age group in Portugal, SINAVE, Portugal March 2020–July 2021. Cases: A total number of cases with a laboratory notification. No CN|EI: cases without clinical notification or epidemic investigation questionnaire submitted. No EI: cases without a submitted epidemic investigation questionnaire overall (regardless of clinical notification).

Those above 75 had a lower prevalence of cases without CN nor EI (31.48%), approximately half of any other age group (Figure 2). We found large differences between regions. Lisbon Algarve and Madeira had a smaller proportion of cases without CN nor EI. Overall, after September 2021, there was an increase in cases without CN nor EI that remained high every month afterward (above 50% in most months until July 2021).

Cases without EI overall were 33.5% in March 2020 and remained below 40% until August 2020. In September, there was an increase in the proportion of cases without EI that remained above 80% after November 2020. Similar to the cases without CN nor EI those aged ≥ 75 had a smaller proportion of cases without EI overall (Tables 1, 2).

**Table 1:** Univariable and multivariable analyses for the outcome No CN nor EI, among laboratory notifications using Poisson regression in Portugal, SINAVE, Portugal March 2020–July 2021.

	**Cases**	**No CN nor EI**	**%**	**aRR**	**[95% Conf. interval]**		***p*-value**
**Sex**							
Female	494,031	265,393	53.72				
Male	415,689	221,058	53.18	0.974	0.971	0.978	<0.001
**Age**							
<25	192,519	116,075	60.29	1	.	.	.
25–49	322,832	184,786	57.24	0.967	0.963	0.972	<0.001
50–74	233,785	135,038	57.76	0.964	0.959	0.968	<0.001
≥75	160,584	50,552	31.48	0.588	0.584	0.593	<0.001
**Region**							
Norte	351,243	219,436	62.47	1	.	.	.
Centro	114,081	70,690	61.96	0.946	0.941	0.95	<0.001
LVT	354,444	146,948	41.46	0.65	0.647	0.653	<0.001
Alentejo	31,904	17,885	56.06	0.87	0.862	0.878	<0.001
Algarve	27,301	10,908	39.95	0.59	0.582	0.598	<0.001
Madeira	10,140	1,222	12.05	0.222	0.211	0.234	<0.001
Açores	6,432	6,012	93.47	1.35	1.34	1.359	<0.001
**Month**							
March-2020	8,124	1,029	12.67	1	.	.	.
April-2020	17,254	2,565	14.87	1.141	1.062	1.225	<0.001
May-2020	8,584	1,252	14.59	1.128	1.035	1.229	0.006
June-2020	10,219	1,251	12.24	1.099	1.011	1.195	0.027
July-2020	9,268	993	10.71	0.972	0.89	1.062	0.527
August-2020	7,243	1,030	14.22	1.238	1.138	1.347	<0.001
September-2020	18,601	5,181	27.85	2.465	2.312	2.627	<0.001
October-2020	68,737	31,318	45.56	3.719	3.501	3.95	<0.001
November-2020	152,790	87,494	57.26	4.505	4.243	4.783	<0.001
December-2020	119,072	61,785	51.89	4.264	4.015	4.528	<0.001
January-2021	305,940	186,496	60.96	5.276	4.969	5.601	<0.001
February-2021	79,310	46,486	58.61	5.404	5.089	5.739	<0.001
March-2021	17,647	8,453	47.9	4.494	4.225	4.779	<0.001
April-2021	13,706	7,113	51.9	4.255	4	4.526	<0.001
May-2021	12,719	7,008	55.1	4.501	4.231	4.788	<0.001
June-2021	32,637	17,870	54.75	4.884	4.597	5.19	<0.001
July-2021	27,809	19,068	68.57	5.883	5.538	6.249	<0.001

**Table 2:** Univariable and multivariable analyses for the outcome without epidemiological investigation questionnaires overall—with or without clinical notification questionnaires among laboratory notifications, using poisson regression in Portugal, SINAVE, Portugal March 2020–July 2021.

	**Cases**	**No EI**	**%**	**aRR**	**[95% Conf. interval]**		***p*-value**
**Sex**							
Female	494,031	414,438	83.89				
Male	415,689	348,074	83.73	0.993	0.991	0.994	<0.001
**Age**							
<25	192,519	169,826	88.21	1	.	.	.
25–49	322,832	280,436	86.87	1	0.998	1.002	0.68
50–74	233,785	204,663	87.54	0.998	0.996	1	0.133
≥75	160,584	107,587	67	0.842	0.839	0.845	<0.001
**Region**							
Norte	351,243	322,459	91.81	1			
Centro	114,081	100,323	87.94	0.905	0.903	0.907	<0.001
LVT	354,444	276,566	78.03	0.828	0.826	0.829	<0.001
Alentejo	31,904	20,439	64.06	0.659	0.654	0.664	<0.001
Algarve	27,301	18,995	69.58	0.705	0.7	0.711	<0.001
Madeira	10,140	3,467	34.19	0.363	0.354	0.373	<0.001
Açores	6,432	6,350	98.73	0.994	0.99	0.998	0.005
**Month**							
March-2020	8,124	2,720	33.48	1	.	.	.
April-2020	17,254	6,878	39.86	1.188	1.146	1.231	<0.001
May-2020	8,584	2,952	34.39	1.037	0.992	1.084	0.105
June-2020	10,219	2,889	28.27	0.894	0.855	0.935	<0.001
July-2020	9,268	2,351	25.37	0.804	0.767	0.843	<0.001
August-2020	7,243	2,780	38.38	1.215	1.165	1.267	<0.001
September-2020	18,601	9,467	50.9	1.597	1.545	1.652	<0.001
October-2020	68,737	49,156	71.51	2.15	2.084	2.217	<0.001
November-2020	152,790	125,657	82.24	2.439	2.365	2.515	<0.001
December-2020	119,072	98,757	82.94	2.537	2.46	2.616	<0.001
January-2021	305,940	291,340	95.23	2.998	2.908	3.092	<0.001
February-2021	79,310	74,940	94.49	3.072	2.979	3.167	<0.001
March-2021	17,647	14,832	84.05	2.856	2.768	2.946	<0.001
April-2021	13,706	11,116	81.1	2.545	2.466	2.627	<0.001
May-2021	12,719	10,617	83.47	2.608	2.527	2.692	<0.001
June-2021	32,637	29,232	89.57	2.865	2.778	2.955	<0.001
July-2021	27,809	26,768	96.26	3.035	2.943	3.13	<0.001

Alentejo, Algarve, and Madeira had the lowest proportions of cases without EI overall as they recovered completeness temporarily after the peak of the second epidemiological wave. Cases aged 75 years or older had a lower proportion without CN or EI (aRR: 0.842 CI95% 0.839–0.845). When compared to the Norte region, cases from Alentejo, Algarve, and Madeira had a lower probability of having no EI (aRR; 0.659 CI 95%0.654–0.664; aRR 0.705 CI 95% 0.7–0.711; and aRR 0.363 CI 95% 0.354–0.373, respectively).

Before January 2021, there was a strong and significant correlation between the number of cases in each month and the proportion of cases without CN nor EI and without EI overall. After January 2021, a reduction in cases was not followed by a reduction or increase in the proportion of cases for either outcome as the Pearson correlation coefficient was low in these periods and not statistically significant (Table 3).

**Table 3:** Pearson correlation coefficient between the number of cases and proportion of cases without CN nor EI (left) and between cases and proportion of cases without EI questionnaires (right) by region in two different periods.

	**Cases/% No CN nor EI**					**Cases/%No EI**				
	≤ **January 2021**		>**January 2021**		**Coef. var**.	≤ **January 2021**		>**January 2021**		**Coef. var**.
	**Pearson coef**	***p*** **-value**	**Pearson coef**	***p*** **-value**		**Pearson coef**	***p*** **-value**	**Pearson coef**	***p*** **-value**	
All regions	0.8736	<0.001	0.3309	0.4685	−0.5427	0.8843	0.0003	0.5484	0.2024	−0.3359
Norte	0.8791	<0.001	0.4189	0.3496	−0.4602	0.8217	0.0019	0.5937	0.1599	−0.228
Centro	0.7795	0.0047	0.2243	0.6287	−0.5552	0.7458	0.0084	0.5835	0.169	−0.1623
LVT	0.9213	<0.001	0.0532	0.9098	−0.8681	0.9173	0.0001	−0.2807	0.542	−1.198
Alentejo	0.9482	<0.001	0.7174	0.0695	−0.2308	0.9434	0	0.7537	0.0504	−0.1897
Algarve	0.9546	<0.001	0.5652	0.1861	−0.3894	0.8759	0.0004	0.7081	0.075	−0.1678
Madeira	−0.2099	0.5355	−0.3273	0.4737	−0.1174	−0.0439	0.8981	−0.12	0.7977	−0.0761
Açores	0.7528	0.0075	0.2169	0.6404	−0.5359	0.3552	0.2837	0.3637	0.4225	0.0085

## Discussion

There has been a very large reduction in the completeness of SINAVE for clinical notification and epidemiological investigation questionnaire submission throughout the pandemic. This reduction started with increasing case numbers after September. We found relevant differences in the outcomes of interest in different age groups, regions, and time periods, which should generate hypothesis on its motifs and contribute to after-action reviews (11) of COVID-19 surveillance and lessons learned for surveillance systems in future pandemics.

There was a correlation between the incidence of cases and the proportion of cases without clinical notification nor EI and without EI questionnaires overall until January. After that, even though case incidence went down, in most regions and age groups, there was only a very mild reduction in these proportions implying changes in the practice of clinicians and public health units that may have prioritized other forms of epidemiological registries.

A lower proportion of cases without CN nor EI in those above 74 can be justified by a higher probability of clinical notification in settings of nursing homes and long-term care facilities because these tend to have closer surveillance by public health units in articulation with the physicians contracted by these institutions.

We can hypothesize that the differences in regions are attributable to different coping capacities with a surge of cases and different strategies adopted toward the use of SINAVE digital platforms for submitting clinical notifications and EI questionnaires during the second wave and after the reduction of cases that started in February 2021. With the rise in cases in October, contact tracing teams were reinforced with professionals from the health sector and others (military, municipal services, etc.), but they may have lacked access to the SINAVE platform. On the other hand, another COVID-19 information system for case management and contact tracing (TRACECOVID-19) had been implemented in April 2020 and acquired new features that generated operational tasks for primary care clinicians (who followed up with patients with COVID-19 by phone) and for public health units (contact tracing association of cases with contacts, contacts follow-up tasks, and automated SMS communication and SARS-CoV-2 test prescrition). Different regions and public health units may have used different platforms to register CN and EI information. All of this may have contributed to an abandonment of clinical notification and EI questionnaires in the SINAVE digital platform, the mandatory, legal framework surveillance system in Portugal.

Even in the first wave, it is apparent that many public health units struggled with submitting EI questionnaires in SINAVE. In the first five pandemic months, even though most laboratory-confirmed cases had a clinical notification, cases without EI questionnaires were above 25%. These values were above 40% in the North region, which implies that in this region, even at the beginning of the pandemic, there was a lower usage of the platform for CN and EI submissions.

The SINAVE digital platform did not allow for the recording of contacts generated by the cases while TRACE COVID-19 came to integrate this function after February 2021, which may have contributed to a shift in platform usage. SINAVE clinical notification included information on testing, symptoms, comorbidities, outcomes, and basic epidemiological context while the EI form included information on (work/institutional/educational context, epidemiological link, origin/context of infection, associated outbreak, travel history, and case classification). Completeness became more limited after October 2021 and especially after January 2020 for most regions and age groups. This has implications in terms of accurate symptom profiling in different epidemiological periods in face of pharmacological and non-pharmacological interventions, in terms of describing the origins and context of infection and unknown sources of infection, in understanding the risk factors over time for different severity outcomes, and in the ability of the surveillance system to measure the impact of changes in policy and of control strategies, including vaccine effectiveness for different outcomes, such as symptomatic disease.

After February 2021, only Algarve and Alentejo had a relevant reduction in the proportion of cases without CN nor EI and cases without EI overall. This may imply that after the surge in demand, these regions made an effort to maintain a high proportion of submitted clinical notifications and EI questionnaires in SINAVE, something that other regions did not do. However, in June and July, even these regions had relevant increases in the proportion of laboratory notifications without clinical notification and epidemiological investigation questionnaires.

These findings should be discussed considering the objectives of the surveillance system and the need for maximizing its usefulness, acceptability, and flexibility in face of evolving epidemiological needs and variations in demand during epidemic surges. The usefulness of specific variables must be evaluated as they should regularly generate useful knowledge for practice and policy. If they do not, they should neither increase the system's burden nor reduce its simplicity and acceptability. Flexibility is also important so that changes are implemented in a fast way to remove unnecessary tasks and variables and add those that become relevant while facilitating operational tasks for data providers at the local-level public health units and healthcare.

We must be aware that surveillance of a pandemic such as COVID-19 is a challenge due to uncertainty and knowledge gaps and due to different information and operational needs that must be anticipated, considering efficiency and acceptability.

Evaluation of surveillance system attributes with a focus on usefulness, while monitoring changes in completeness, timeliness, and data quality over time is of utmost importance. It allows anticipation of the need to change or integrate surveillance, to increase acceptability by maximizing efficiency, and to give feedback to the operational level while guaranteeing that the system is serving its objectives. Usefulness should be maximized in terms of operational management of cases and outbreaks, and in terms of generating quality data to inform decisions and allow applied epidemiological research beyond surveillance routine outputs to further understand the disease and inform policy and practice.

However, SINAVE-specific surveillance and research objectives for COVID-19 have not been publicly and formally defined even though SINAVE legal and technical framework defines general objectives for all notifiable diseases (1).

These findings must raise awareness of the potential of pandemics to disrupt surveillance systems and induce changes in their use, with consequences in terms of the availability of centralized and complete data. We also raise awareness of the importance of frequent evaluation and monitoring of the performance of the surveillance systems considering objectives and usefulness, both operational (case and outbreak investigation and transmission control) and strategic (information for policy). Evaluation of various attributes of surveillance systems for COVID-19 and other pandemics must be promoted to inform and anticipate the necessary changes in surveillance systems architecture and procedures and maximize their value.

Other evaluations of surveillance systems for COVID-19 suggest that public health institutes should improve physicians' and public health units' participation in the system by improving access to data, providing support and feedback that is useful in daily work in the field and that evaluation of surveillance systems for COVID-19 should be conducted in a systematic way (5).

To our knowledge, few studies have analyzed the completeness of clinical and public health notification components of surveillance among all laboratory-confirmed cases in a country for COVID-19.

These findings have implications in terms of the cost-effectiveness of surveillance systems. Concrete public health action and policy were less robustly supported by SINAVE due to the low completeness of clinical notification and EI questionnaires. Other COVID-19 information systems such as TRACE-COVID-19 could be considered in terms of defining the future approach to COVID-19 surveillance in Portugal. Patients autonomous submission of relevat clinical and epidemiological information should be considered in different contexts.

Low completeness of surveillance systems and gaps in collected information weakens health policy and practice, understanding of transmission patterns, clinical presentation, and risk factors for infection and disease severity over time, and evaluation of the impact of control interventions (contact tracing, vaccination, and others).

Improvements to surveillance systems must be driven by public health objectives and should be evaluated rigorously to determine their effects and costs. These objectives need to be clear and fully described to evaluate whether the system remains fit for purpose over time.

For COVID-19 as well as future pandemic threats and other notifiable diseases in Portugal, it is important to advance toward one centralized, robust surveillance system that can gather all relevant data (linking different platforms and health registries (primary care, hospitals, vaccination registries, contact tracing platforms, and prescriptions) to maximize usefulness, simplicity, and acceptability, considering different objectives and stakeholders.

Routine monitoring of system performance and conduction of evaluations of the surveillance system is of utmost importance in pandemics. The surveillance system's attribute indicators should be closely monitored, reported, and discussed considering information needs and knowledge gaps in different pandemic periods. Pandemics have a large impact on health, society, and economy, and knowledge of surveillance systems functioning and attributes is critical to facilitate planning, propose changes, and interpret surveillance findings adequately (12).

## Data availability statement

The data analyzed in this study is subject to the following licenses/restrictions: DGS Portugal COVID-19 Surveillance SINAVE Dataset. It's been shared with the academia under a protocol during the COVID-19 pandemic. Requests to access these datasets should be directed to DGS, Portugal.

## Ethics statement

Ethical review and approval was not required for this study of routine, anonymized surveillance data, in accordance with the local legislation and institutional requirements. Written informed consent for participation was not required for this study in accordance with the national legislation and the institutional requirements.

## Author contributions

VR: design, analysis, and writting of first draft. AV: reviews. PA: desing, analysis, and reviews. AA: design and reviews. DR, CC, AS, and CN: discussion and reviews. All authors contributed to the article and approved the submitted version.
